# Endothelia-Targeting Protection by Escin in Decompression Sickness Rats

**DOI:** 10.1038/srep41288

**Published:** 2017-01-23

**Authors:** Kun Zhang, Zhongxin Jiang, Xiaowei Ning, Xuhua Yu, Jiajun Xu, Peter Buzzacott, Weigang Xu

**Affiliations:** 1 Department of Diving and Hyperbaric Medicine, Faculty of Naval Medicine, the Second Military Medical University, Shanghai, China; 2 School of Sports Science, Exercise and Health, the University of Western Australia, Perth, Australia

## Abstract

Endothelial dysfunction is involved in the pathogenesis of decompression sickness (DCS) and contributes substantively to subsequent inflammatory responses. Escin, the main active compound in horse chestnut seed extract, is well known for its endothelial protection and anti-inflammatory properties. This study aimed to investigate the potential protection of escin against DCS in rats. Escin was administered orally to adult male rats for 7 d (1.8 mg/kg/day) before a simulated air dive. After decompression, signs of DCS were monitored, and blood and pulmonary tissue were sampled for the detection of endothelia related indices. The incidence and mortality of DCS were postponed and decreased significantly in rats treated with escin compared with those treated with saline (*P* < 0.05). Escin significantly ameliorated endothelial dysfunction (increased serum E-selectin and ICAM-1 and lung Wet/Dry ratio, decreased serum NO), and oxidative and inflammatory responses (increased serum MDA, MPO, IL-6 and TNF-α) (*P* < 0.05 or *P* < 0.01). The results suggest escin has beneficial effects on DCS related to its endothelia-protective properties and might be a drug candidate for DCS prevention and treatment.

Decompression sickness (DCS) is associated with the genesis of inert gas bubbles which grow from pre-existing gas micronuclei in supersaturated tissues and blood during or after a rapid drop of ambient pressure^1^. It is now widely accepted that DCS is a systemic pathophysiological process^2^. A number of studies report that endothelial activation and dysfunction occurred after decompression, followed by synthesis of cytokines and cell adhesion stimulators, and finally systematic inflammation^2^,^3^,^4^,^5^. We recently published a study showing that decompression-induced endothelial dysfunction correlated well with bubble formation in a rat model^6^, and another study by us revealed that simvastatin significantly reduced DCS injury in rats^7^, and short-acting nitric oxide (NO) donors also proved to have protective effects against DCS in pigs^8^. The beneficial effects of these agents on DCS may, at least partially, be attributed to their endothelia-protective properties. We hypothesized that endothelial protective drugs may alleviate the severity of DCS.

Escin, or aescin, a natural mixture of triterpene saponins, is the major active compound in extracts from the seeds of Aesculus hippocastanum (horse chestnut tree), a widely used medicinal plant since ancient times^9^,^10^. It exists in two forms, α and β and the latter is the active component of the mixture^10^. Escin has been traditionally used as a carminative, stomachic, and endothelia-protective agent in treating peripheral vascular disorders, in particular varicosis^11^. It is recognized as having vasoconstrictor activity by increasing sensitivity to molecular ions, resulting in increased venous tension^11^,^12^. Accumulating evidence suggests that escin exerts anti-edematous and anti-inflammatory properties mainly due to its endothelia-protective ability^11^. Escin reduces endothelia activation and sensitization to ions resulting in decreased neutrophil adherence and permeability to water^13^, and exerts anti-inflammatory and synergistic anti-inflammatory effects without immunosuppression^10^,^14^. In addition, escin has been found effective as an antioxidant agent with the ability to scavenge reactive oxygen species (ROS)^15^ and induce endothelial NO synthesis by sensitization to Ca^2+^ ions^16^.

The purpose of this study was to assess the effect of escin on a DCS rat model and to explore the mechanisms underlying observed effects. The incidence and mortality of DCS, and endothelia related indices, were determined.

## Methods

### Animals

A total of 90 adult male rats (Sprague-Dawley strain) were obtained from Shanghai Slac Laboratory Animal Co. Ltd. The experiment protocol was approved by the Animal Ethics Committee of the Second Military Medical University and the procedures were carried out in compliance with related guidelines and regulations. Animals were maintained in the laboratory under a controlled environment with a 12/12-h light/dark cycle and allowed *ad libitum* access to a pelleted rodent diet and water during the experiment. Temperature was maintained at 23 ± 1 °C and relative humidity at 54 ± 2%.

### Procedure and design

The rats were randomly divided into three groups: 40 each for the Escin and Saline group, and 10 for the Normal control group. The sample size was estimated according to our preliminary experiment, which indicated that the incidence of DCS would be around 75% for Saline rats and 45% for Escin rats. It was calculated that 40 rats per group would provide 80% power showing a significant difference in the incidence based on a two-tailed significance level of 0.05. Rats in the former two groups were subjected to a simulated air dive in a chamber to induce DCS. Normal rats were sham exposed (normobaric air) in the same chamber and used to acquire normal values of the indices. Rats in the Escin group were treated for 7 d by oral gavage with sodium β-escin (Sigma-Aldrich, Toluca, Mexico) 1.8 mg/kg body weight daily dissolved in physiological saline. Rats in the Saline group received the same volume of physiological saline without escin. Because body weight significantly affects bubble formation and DCS incidence in rats, the weight of all rats at the time of DCS modeling was strictly controlled within a narrow range of 300–310 g by starting administration at the weight 265–275 g. Following rapid decompression, the rats were observed for 30 min for DCS diagnosis by a member of staff blinded to the drug arm conditions. Surviving rats were anesthetized with 3% pentobarbital sodium (1.5 ml/kg body weight, i.p.) 2 h after decompression. Blood and tissues were then sampled for biochemical analysis. Normal control rats were similarly sampled.

### Simulated diving

Rats in the escin and saline treated groups were compressed with air in a transparent hyperbaric rodent chamber (Type RDC150-300-6, SMMU, Shanghai, China) in pairs, each time with one in the Escin and the other in the Saline group. The pressure was raised to 7 atmospheres absolute (ATA) in 5 min which began at a low rate (0.5 ATA/min) to minimize possible middle ear squeeze and then maintained for 90 min. Thereafter, decompression was carried out linearly to ambient pressure at a rate of 2 ATA/min. The chamber was ventilated continuously with compressed air during the exposure to avoid carbon dioxide (CO_2_) retention.

### DCS symptoms observation

Following decompression the rats were subjected to walk inside an electrically controlled cylindrical cage rotating at a perimeter speed of 3 m/min for 30 min to standardize activity level and facilitate DCS diagnosis. According to our previous studies, 30 min of observation was long enough for all cases of DCS to become evident^7^,^17^. Any of the following symptoms were considered manifestations of DCS: respiratory distress, walking difficulties, forelimb and/or hindlimb paralysis, rolling in the rotating wheel, convulsions or death^7^. Death is commonly used as a parameter of DCS in rat model experiments^7^,^17^ and the animal ethics committee approved death as one of the endpoints in this study. As all the survived DCS rats recovered at 5–15 min after the initial occurrence of symptoms, and as death cases occurred either suddenly without obvious signs or following a brief period of convulsions, no rats suffered severe pain. A double classification was applied differentiating: “no DCS” and “DCS”, and whenever the symptoms described above (including death) were observed then latency to DCS symptoms was also recorded.

### Assay of serum biochemical indices

Venous blood was drawn from the right ventricle under anesthesia. Serum levels of E-selectin, intercellular cell adhesion molecule-1 (ICAM-1), interleukin-6 (IL-6), tumor necrosis factor-α (TNF-α) were assayed by ELISA (Jiancheng Bioengineering Institute, Nanjing, China). Nitric oxide (NO) was measured using the Griess reaction and malondialdehyde (MDA) was detected by thiobarbituric acid colorimetric methods, both using commercial assay kits (Beyotime Institute of Biotechnology, Nantong, China). The enzyme activity of myeloperoxidase (MPO) and superoxide dismutase (SOD) were determined by ELISA using the respective assay kits (Meilian Biological Technology Co, Shanghai, China). All assays were performed following the respective manufacturer’s instructions.

### Lung Wet/Dry weight ratio assay

The severity of pulmonary edema was estimated by the wet-to-dry (W/D) lung weight ratio. The weight of the lung tissue was determined from the right lung lobes. The fresh tissue was weighed (wet weight), incubated at 120 °C for 3 days and then weighed again (dry weight).

### Statistical analysis

Except for incidence and mortality, all data are presented as mean ± SD. Incidence and mortality of DCS after decompression were compared between the Escin and Saline group by Chi-square test. Differences in latency to symptoms and in survival were analyzed with the log-rank test. Continuous variables were tested for normal distribution with the Kolmogorov-Smirnov test and were compared by one-way ANOVA followed by Student-Newman–Keuls tests or post hoc Dunnett’s tests among Escin, Saline and Normal groups. The threshold for significance was accepted at *P* < 0.05.

## Results

### The incidence and mortality of DCS

**Figure 1:**
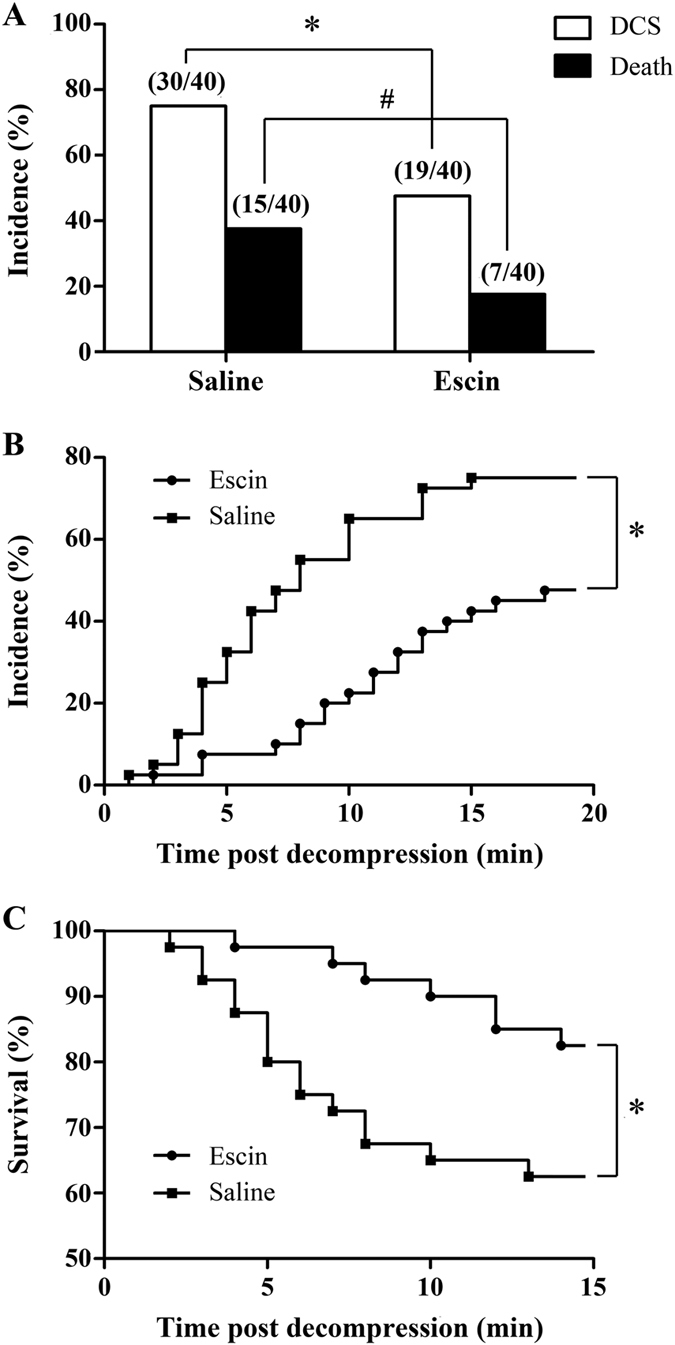
Effects of escin on the incidence and mortality of DCS in rats. Rats were diagnosed during the 30 min period walking on a rotating cylindrical cage after rapid decompression from a simulated air dive to 7 ATA for 90 min. (**A**) The incidence and mortality of DCS in rats treated with escin or saline, *P = 0.012, ^#^*P* = 0.045. (**B**) The incidence of DCS with time, *P = 0.001. (**C**) Survival curve of rats, *P = 0.003.

The incidence and mortality of DCS in the Escin group were significantly lower than in the Saline group (75% *vs.* 47.5%, *P* = 0.012 and 37.5% *vs.* 17.5%, *P* = 0.045; Fig. 1A). All cases of DCS and death occurred within 18 min after surfacing (Fig. 1B and C). The majority of deaths occurred suddenly without obvious signs or preceded by a short period of convulsions lasting 5–10 seconds. As shown in Fig. 1B and C, the latency to DCS in the Escin group was significantly delayed (*P* = 0.001) and survival time also significantly differed between the two groups (*P* = 0.003).

### Effects of escin on endothelial indices in DCS

**Figure 2:**
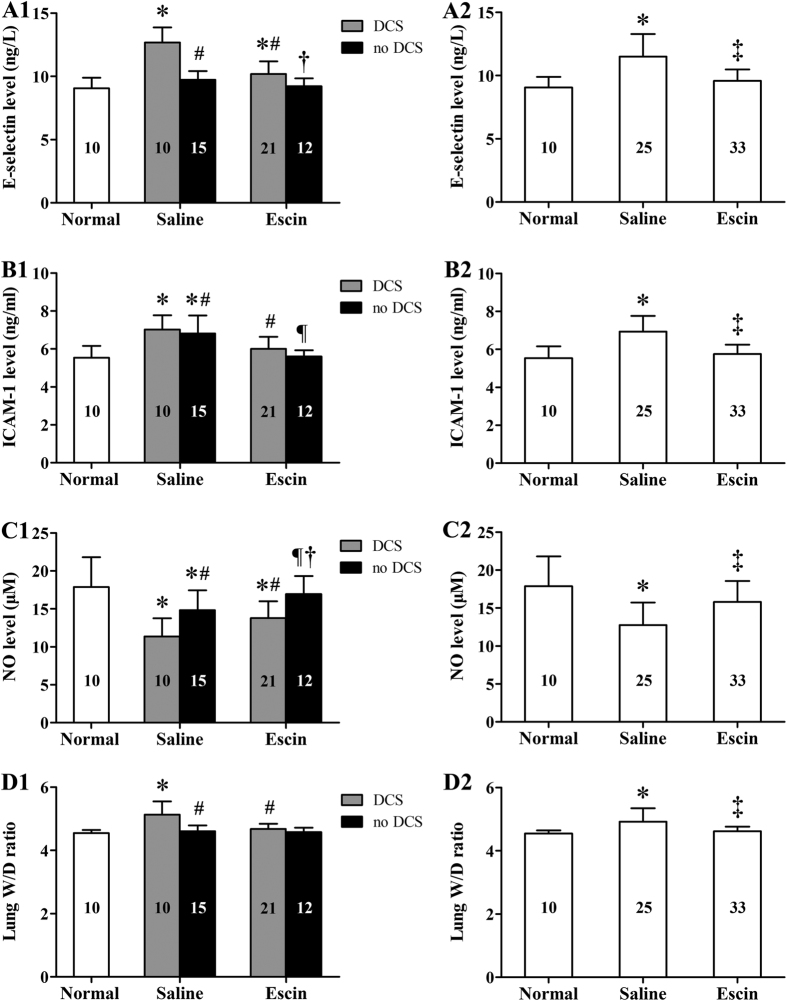
Effects of escin on DCS-induced changes in endothelial biomarkers in rats. The parameters were determined 2 h after decompression from a simulated air dive. All surviving rats were sampled. Figure A1–D1 present by DCS status, Figure A2–D2 show whole groups. The number of animals in each group is listed in the column. *P < 0.05 *vs.* Normal controls, ^#^*P* < 0.05 *vs.* Saline DCS, ^¶^*P* < 0.05 *vs.* Saline no DCS, ^†^*P* < 0.01 *vs.* Escin DCS, ^‡^*P* < 0.01 *vs.* Saline rats.

Endothelial function was assessed by examining serum levels of E-selectin, ICAM-1, NO and lung W/D ratio. As shown in Fig. 2, serum levels of E-selectin and ICAM-1 and lung W/D ratio increased, serum NO concentration decreased significantly in Saline rats either among those suffering DCS (shown as Figure A1–D1) or in the group as a whole (shown as Figure A2–D2) when compared with Normal rats (*P* < 0.01). Escin treatment reversed these changes (*P* < 0.05). ICAM-1 and NO changed significantly in rats with no DCS in the Saline group (*P* < 0.01), also reversed by escin (*P* < 0.01).

### Anti-oxidative effects of escin in DCS

**Figure 3:**
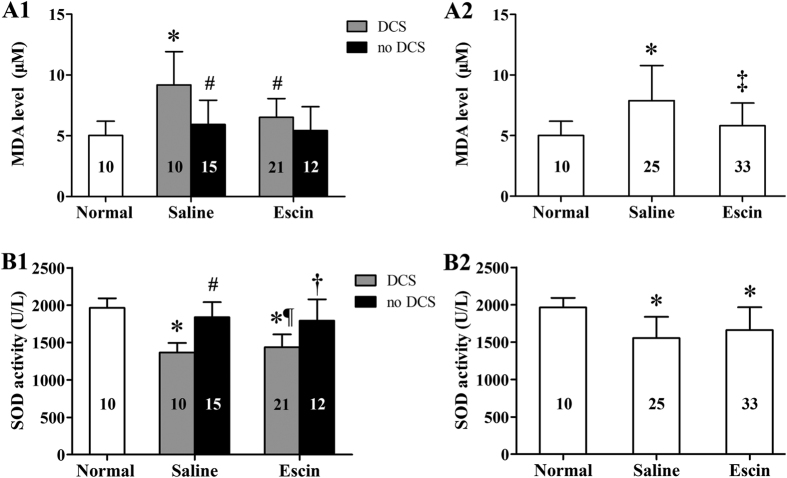
Effects of escin on serum level of MDA and SOD activity in DCS rats. Serum MDA level and SOD activity were detected in surviving rats 2 h after rapid decompression from a simulated dive to 7 ATA for 90 min. Figure A1–B1 present by DCS status, Figure A2–B2 show whole groups. The number of animals in each group is listed in the column. *P < 0.01 *vs.* Normal controls, ^#^*P* < 0.05 *vs.* Saline DCS, ^¶^*P* < 0.01 *vs.* Saline no DCS, ^†^*P* < 0.05 *vs.* Escin DCS, ^‡^*P* < 0.05 *vs.* Saline group.

Oxidative stress and antioxidative capacity were assessed by examining serum MDA level and SOD activity (Fig. 3). In saline treated rats suffering DCS or in the group in total, MDA levels increased and SOD activity decreased significantly compared with Normal controls (*P* < 0.01 for either). Escin treatment reduced the increased MDA (*P* = 0.036) but did not change SOD activity (*P* = 0.924) in DCS rats. Significant differences were found between rats with and without DCS in three subgroups (*P* < 0.05) except for MDA in the Escin group (*P* = 0.563).

### Anti-inflammatory effects of escin in DCS

**Figure 4:**
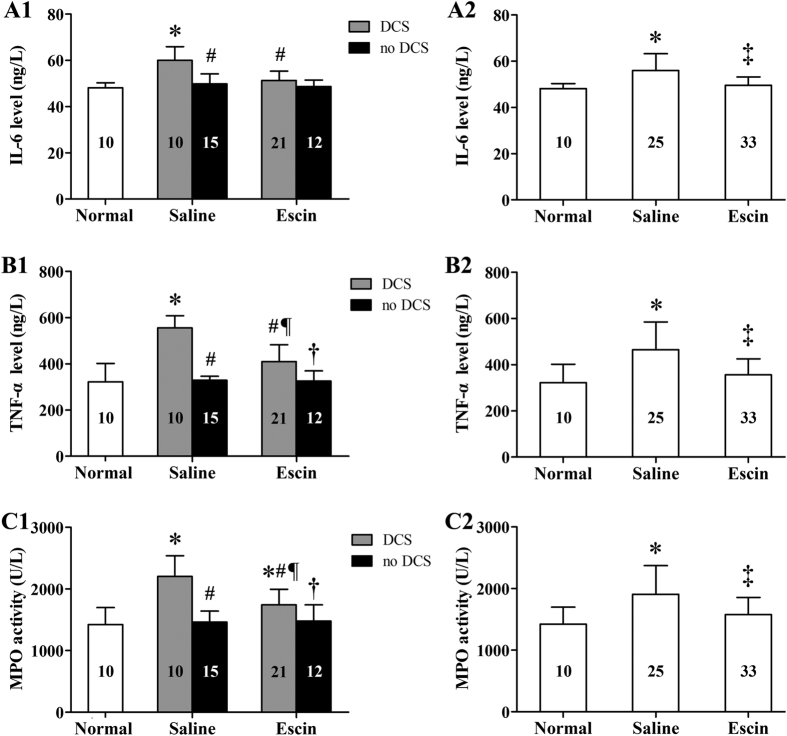
Effects of escin on inflammatory responses in DCS rats. The parameters were determined at 2 h after decompression from a simulated diving to 7 ATA for 90 min in all survived rats. Figure A1–C1 present by DCS status, Figure A2–C2 show whole groups. The number of animals in each group is listed in the column. *P < 0.01 *vs.* Normal controls, ^#^*P* < 0.01 *vs.* Saline DCS, ^¶^*P* < 0.05 *vs.* Saline no DCS, ^†^*P* < 0.01 *vs.* Escin DCS, ^‡^*P* < 0.05 *vs.* Saline group.

Changes of circulating cytokines and MPO activity are shown in Fig. 4. Serum IL-6 and TNF-α levels and MPO activity in Saline rats suffering DCS or in the group as a whole increased significantly compared with Normal controls (*P* < 0.05), and escin reduced these changes (*P* < 0.05). There were significant differences between rats with and without DCS in either group (*P* < 0.05) except for IL-6 in escin treated rats (*P* = 0.427).

## Discussion

DCS is a concern for people who take part in recreational or professional diving, harvesting from the sea, hyperbaric medicine or spaceflight. It occurs following a sudden or excessively rapid ascent or even after well-controlled decompression^1^. Vascular endothelial cells are vulnerable to dive decompression, and mechanical damage is most likely one of the main triggers of the inflammatory cascade in DCS etiology^2^,^3^. Circulating bubbles, oxidative stress and other diving-induced insults are thought involved in the pathogenesis of endothelial dysfunction^3^,^18^,^19^. Potential preventative and therapeutic approaches against this pathogenesis have been persistently explored^7^,^8^,^17^,^19^, but no studies investigating approaches that directly protect endothelia in DCS pathology have been reported.

Although much recent attention focuses on its anti-cancer properties, escin is well known for its remarkable efficacy in treating chronic venous insufficiency (CVI), hemorrhoids and post-operative edema^11^. Therapeutic benefit is supported by a number of investigations using various models and clinically, indicative of clear endothelia-protective effects, most of which appear due to selective endothelial permeabilization to ions and other molecules^9^. These sensitizing effects make escin capable of maintaining adherent junction integrity between endothelial cells, causing a ‘sealing effect’ in small vessels permeable to water, increasing endothelial nitric oxide synthase (eNOS) activity, and finally preserving endothelial integrity and function^13^,^15^. Increased eNOS was a critical factor in protecting endothelial function, facilitating the elimination of vascular gaseous nuclei and thereby may reduce the bubble formation^20^. Escin may inhibit the activation of endothelial cells and the subsequent secretion of proinflammatory mediators like E-selectin and adhesion molecules in thrombotic events or gas embolism^13^,^16^,^21^. Other mechanisms, including protection of the reorganization of endothelial cytoskeleton, and inhibition of the enzymes elastase and hyaluronidase, further underline the wide ranging mechanisms of the endothelia-protective activity of escin^9^,^11^.

Concerning the role of endothelial injury in the pathophysiology of DCS, the potential protective effects of escin on DCS was investigated in this study. Intravenous injection of escin may cause phlebitis or other adverse reactions involving multiple organs and systems, while oral administration can avoid these side effects within the clinical dosing range 5–20 mg/d^22^. The dosage utilized in the present study (1.8 mg/kg body weight daily) was well within the range of human dosing regimens in accordance with the formula of conversion between different species^23^ and no side effects were observed. Among the determined parameters, lung W/D weight ratio is an indicator of lung microvascular permeability, E-selectin and ICAM-1 are sensitive biomarkers with the capacity to reflect endothelial damage, and NO is a vasoactive substance secreted by endothelial cells. Escin effectively counteracted endothelium related injuries in DCS rats, and showed potential to reduce both incidence and mortality of DCS. For the rats spared DCS, only two of the nine parameters determined showed significant changes, and these were also reversed by escin.

The preventative effects of escin on DCS were also likely related to its anti-inflammatory properties, which may still rest partly on its endothelial protective effects. Escin has been shown to be effective in interfering with the cellular phase of the inflammatory process^9^. As described above, escin can inhibit the activation of endothelial cells and subsequent adhesiveness of neutrophils, inhibiting the release of cytokines and inflammatory mediators^13^,^24^. In the present study, levels of IL-6, TNF-α and MPO activity, which reflect neutrophil infiltration following endothelial injury, were significantly decreased in escin-treated DCS rats. Escin also exerts an anti-inflammatory effect by enhancing the release of glucocorticoids (GC), elevating GC receptor expression, decreasing histaminic activities and nuclear factor-κ B activation^14^,^25^, which may also play a role in protection against DCS.

In addition to endothelial protection and anti-inflammation, other properties of escin may also mitigate DCS. Accumulating evidence suggests that oxidative stress plays an important role in the pathogenesis of DCS, and antioxidants significantly reduce DCS injury including endothelial dysfunction^17^,^19^,^26^. Escin has the potential to act as a free radical scavenger through its hydrogen-donating and ion-chelating capability due to the presence of hydroxyl groups in the molecule^15^. These anti-oxidative effects may play a role in escin’s endothelial protective properties by scavenging reactive oxygen species (ROS) which cause depletion of NO^11^. Escin treatment showed obvious suppressive effects on the elevation of MDA induced by decompression stress. However, though it reduced MDA, escin controversially failed to reverse a decrease in SOD activity in two previous studies^27^,^28^.

DCS may occur without evident violations of diving protocols and is hard to predict, therefore protective therapeutic approaches could prove to be of great benefit^29^. The endothelia-protective, anti-oxidative and anti-inflammatory properties of escin make it an ideal candidate for pre-treating against DCS. For the rat DCS model adopted in this study, all signs of DCS occurred shortly after rapid decompression, while escin reaches maximal levels in plasma after approximately 3 h following oral administration and has a half-time for disappearance around 8–12 h^30^. Pre-dive administration of escin therefore potentially facilitates prevention over post-DCS administration for treatment. In light of such considerations, escin was administered for a week prior to the simulated dive, which increased the possibility of observing beneficial effects. However, administration after DCS is postulated to also exert therapeutic effects during the long pathophysiological process of DCS in divers. This possibility remains to be tested. The vasoconstrictor property of escin which may result in limiting inert gas loading during hyperbaric exposure, and delaying the removal during and after decompression, may have paradoxical effects on its protection against DCS^31^. Although in the present study no adverse effect was observed, the contractile effect of escin should be considered before recommending escin for DCS prevention.

In this study, biochemical parameters were separately observed in rats with or without DCS. All determined biochemical indices changed significantly in rats suffering DCS in the Saline group, and only NO and ICAM-1 significantly changed in rats with no DCS. Escin prevented all these changes, indicating high potential efficacy in DCS prevention. Meanwhile, these results also provide evidence that changes in biochemical indices correlate with DCS severity. When combining the results from DCS and no DCS rats, SOD showed no significant changes in the Escin group. This indicates that the results from sub-groups of DCS rats could reveal more information than by considering groups of rats receiving treatment as a whole. However, observing no visible signs of DCS does not necessarily mean no changes occured in biochemical indices.

In conclusion, this is the first study to explore the effects of escin on DCS. Oral administration of escin exerted significant protection against DCS in rats, mostly through endothelial protective and anti-inflammatory properties. Besides identifying the beneficial effects of escin on DCS, the present results further support that endothelial dysfunction plays an important role in the pathophysiology of DCS and, therefore, endothelial protection could indeed alleviate DCS injuries. Further explorations of endothelial protective agents on preventing or treating DCS are encouraged. As a well-used agent clinically, escin is safe and may be readily adopted in preventing or treating DCS if supported by necessary clinical trials.

## Additional Information

**How to cite this article**: Zhang, K. *et al*. Endothelia-Targeting Protection by Escin in Decompression Sickness Rats. *Sci. Rep.* **7**, 41288; doi: 10.1038/srep41288 (2017).

**Publisher's note:** Springer Nature remains neutral with regard to jurisdictional claims in published maps and institutional affiliations.
